# Simulated 50 % radiation dose reduction in coronary CT angiography using adaptive iterative dose reduction in three-dimensions (AIDR3D)

**DOI:** 10.1007/s10554-013-0190-1

**Published:** 2013-02-13

**Authors:** Marcus Y. Chen, Michael L. Steigner, Steve W. Leung, Kanako K. Kumamaru, Kurt Schultz, Richard T. Mather, Andrew E. Arai, Frank J. Rybicki

**Affiliations:** 1 Advanced Cardiovascular Imaging Laboratory, Cardiovascular and Pulmonary Branch, Department of Health and Human Services, National Heart, Lung and Blood Institute (NHLBI), National Institutes of Health (NIH), Bethesda, MD USA; 2 Applied Imaging Science Laboratory, Department of Radiology, Brigham and Women’s Hospital, Harvard Medical School, Boston, MA 02115 USA; 3 Toshiba Medical Research Institute, 706 N Deerpath Dr, Vernon Hills, IL USA

**Keywords:** Coronary imaging, Computed tomography, Angiography, Image reconstruction, Radiation dose

## Abstract

To compare the image quality of coronary CT angiography (CTA) studies between standard filtered back projection (FBP) and adaptive iterative dose reduction in three-dimensions (AIDR3D) reconstruction using CT noise additional software to simulate reduced radiation exposure. Images from 93 consecutive clinical coronary CTA studies were processed utilizing standard FBP, FBP with 50 % simulated dose reduction (FBP50 %), and AIDR3D with simulated 50 % dose reduction (AIDR50 %). Signal-to-noise ratio (SNR) and contrast-to-noise ratio (CNR) were measured within 5 regions-of-interest, and image quality for each reconstruction strategy was assessed by two independent readers using a 4-point scale. Compared to FBP, the SNR measured from the AIDR50 % images was similar or higher (airway: 38.3 ± 12.7 vs. 38.5 ± 14.5, *p* = 0.81, fat: 5.5 ± 1.9 vs. 5.4 ± 2.0, *p* = 0.20, muscle: 3.2 ± 1.2 vs. 3.1 ± 1.3, *p* = 0.38, aorta: 22.6 ± 9.4 vs. 20.2 ± 9.7, *p* < 0.0001, liver: 2.7 ± 1.0 vs. 2.3 ± 1.1, *p* < 0.0001), while the SNR of the FBP50 % images were all lower (*p* values < 0.0001). The CNR measured from AIDR50 % images was also higher than that from the FBP images for the aorta relative to muscle (20.5 ± 9.0 vs. 18.3 ± 9.2, *p* < 0.0001). The interobserver agreement in the image quality score was excellent (*κ* = 0.82). The quality score was significantly higher for the AIDR50 % images compared to the FBP images (3.6 ± 0.6 vs. 3.3 ± 0.7, *p* = 0.004). Simulated radiation dose reduction applied to clinical coronary CTA images suggests that a 50 % reduction in radiation dose can be achieved with adaptive iterative dose reduction software with image quality that is at least comparable to images acquired at standard radiation exposure and reconstructed with filtered back projection.

## Introduction

Cardiac computed tomography angiography (CTA) is an established non-invasive method to evaluate the coronary arteries [1] with a high negative predictive value to exclude significant coronary artery disease [2]. Radiation exposure is a concern due to the potential for an increased lifetime risk of malignancy [3], and thus technology and practice patterns have evolved to utilize more prudent image acquisition techniques with respect to radiation exposure [4].

Recent advances in computing power and algorithm optimization have enabled clinical use of iterative reconstruction methods [5, 6] with improvements in image quality and/or reduction of radiation dose [7–13]. Iterative reconstruction algorithms that use raw, projection data are vendor specific with software that is proprietary to the individual CT manufacturer. A new Adaptive Iterative Dose Reduction (AIDR) algorithm in Three-Dimensions (AIDR3D) works in both the raw and image domains and is fully integrated into the 320 × 0.5 mm detector row CT acquisition workflow.

Prior studies using AIDR, a precursor to AIDR3D, demonstrate improved image quality using an iterative reconstruction [14, 15]. Another study showed the improvement in image quality using AIDR3D compared to FBP and first-generation AIDR in low dose chest CT [16]. Iterative reconstruction has been applied to 320 × 0.5 mm detector row cardiac CT with a reduction of image noise [17, 18]. These studies did not suggest a specific reduction of patient radiation exposure. Another study looked at two different patient groups, one before the inclusion of AIDR3D and one including AIDR3D with a moderately lower mA [19]. However, none of these studies have examined the dose reduction effects of AIDR3D in a single patient group. An assessment of the benefits and potential tradeoffs of applying iterative reconstruction for clinical coronary CTA imaging be estimated, without repeating clinical scanning, by mathematically adding CT noise to the sinogram data to simulate reductions in tube current. The purpose of this study is to test the hypothesis that the AIDR3D reconstruction will (a) reduce the magnitude of noise as measured by clinical regions of interest and (b) maintain image quality for clinical coronary CTA images reconstructed with a simulated 50 % reduction in tube current.

## Subjects and methods

### Demographics

The study was approved by the institutional review board at two institutions. A database of 93 subjects was created from pooling 52 consecutive clinical CTA exams from institution 1 with 41 consecutive CTA exams from institution 2. Baseline characteristics (Table 1) were obtained from the hospital electronic medical records of both institutions.

**Table 1:** Patient demographics

Age (year)	51.5 ± 14.4 (15–84)
Gender (M:F)	59:34
Weight (kg)	77.3 ± 16.9 (49–133)
BMI (kg m^−2^)	26.6 ± 4.8 (18–42)
Heart rate at CT scan (bpm)	56.3 ± 6.5 (42–80)
Iodinated contrast amount (ml)	67.2 ± 7.6 (50–80)
Effective dose (mSv, k = 0.014 mSv mGy^−1^ cm^−1^)^a^	4.3 (1.7–6.4)
Clinical indication	
Chest pain	57
Pre-operative evaluation	9
Known CAD or post-PCI	7
Known other coronary diseases^b^	6
Heart failure	5
Equivocal or abnormal stress test	5
Anomalous coronary artery	4

Continuous values expressed as mean ± SD (range)

*CAD* coronary artery disease, *PCI* percutaneous coronary intervention

^a^Expressed as median (interquartile range)

^b^Fistula, dissection, and aneurysm

### CT scan parameters

All patients were imaged with axial 320 × 0.5 mm detector row CT [20, 21] (AquilionONE, Toshiba Medical Systems Corporation, Japan) using asymmetric cone beam reconstruction [22]. All scanning was done within 1 heart beat using prospective ECG gating except for one subject who underwent 2-beat retrospective ECG-gated CCTA for an evaluation of cardiomyopathy. The gantry rotation was 350 ms; images were reconstructed at 0.5 mm increments. The kV and mAs were chosen by the attending cardiovascular imager and were largely determined by patient body habitus. Overall, 52 % of the studies were imaged at 100 kV and the remaining 48 % were imaged at 120 kV. Iopamidol 370 mg iodine/mL (Isovue 370, Bracco Diagnostics, Princeton, NJ) was injected via an antecubital intravenous line at an injection rate of 5–6 ml per second. The contrast volume (50–80 ml) was based on body habitus and determined by the attending cardiovascular imager. Contrast enhanced images were timed with bolus tracking within the descending aorta at a trigger threshold value of 200 HU (institution 1) or 180 HU (institution 2). Patients received oral and/or IV β-blockade (metoprolol, 5-mg increments to a maximum of 30 mg) at the discretion of the attending cardiovascular imager. Patients also received 0.4 mg of sublingual nitroglycerin for coronary vasodilation.

### Noise simulation

Sinogram data was retrieved from the scanner systems and archived using a raw data server (Toshiba Medical Systems Corporation, Japan) equipped to add noise to the sinograms with a noise addition tool. Both the raw data server and the noise addition software were used under a research agreement with the manufacturer. The tool models specific system noise empirically with water phantom scans at various acquisition settings and injects a combination of Poisson noise for photon statistics and Gaussian electronic noise into the raw projections based on the desired reduction in tube current to be simulated.

### Image data reconstruction

Each raw data was reconstructed three times: the first reconstruction (termed “FBP”) used the original acquired raw data and the manufacturer filtered back projection (FC03) kernel at the 75 % phase of the R–R interval. The second reconstruction (termed “FBP50 %”) was identical to the first except for the fact that CT noise was added to the raw data to simulate a 50 % reduction in mAs before the FC03 kernel was applied. The third reconstruction (termed “AIDR50 %”) used the standard AIDR3D after the raw data underwent the same simulated 50 % reduction in mAs.

### Image quality and dose estimation

To compare attenuation and image noise between the three reconstructed data sets, region-of-interest (ROI) measurements of mean and standard deviation (SD) of Hounsfield Units (HU) were obtained in the descending aorta, trachea, pectoral muscle, the fat superficial to the pectoral muscle, and liver parenchyma. Signal-to-noise ratio (SNR) was calculated by dividing the absolute mean value within the region of interest by its SD. To compare contrast-to-noise ratio (CNR) between the three image data sets, three contrasts were measured as difference between the two mean CT numbers divided by the SD of the organ of interest. The first was between the descending aorta and the pectoral muscle. The second was between the pectoral muscle and fat. The third was between the pectoral muscle and the trachea. Pectoral muscle measurements were used as a surrogate for myocardium due to beam-hardening artifacts frequently observed within the myocardium [23]. Patient effective radiation doses were estimated using the dose length product reported by the scanner and a conversion factor of k = 0.014 mSv/mGy-cm [24].

### Qualitative analysis

To evaluate potential differences between the images reconstructed with filtered back projection versus iterative reconstruction, all image data sets were anonymized, randomized, and transferred to an image post-processing workstation (Vitrea FX, Vital Images, Minnetonka, MN, USA). Two experienced cardiovascular imagers (one from each institution) blinded to the acquisition and reconstruction technique independently evaluated overall image quality using a 4-point scale based on vessel sharpness, image noise, streak or other artifacts where 4 = excellent, no artifact; 3 = good, mild artifact; 2 = acceptable, moderate artifact present but images still interpretable; and 1 = unevaluable with severe artifacts rendering interpretation not possible. Additionally, all 279 datasets were independently evaluated by these two readers for the presence of obstructive coronary artery disease (positive if at least one segment had ≥50 % luminal stenosis); this evaluation was blinded to clinical information and reconstruction method. Discrepancies in scores and clinical interpretation were adjudicated by joint consensus reading.

### Phantom study

**Fig. 1:**
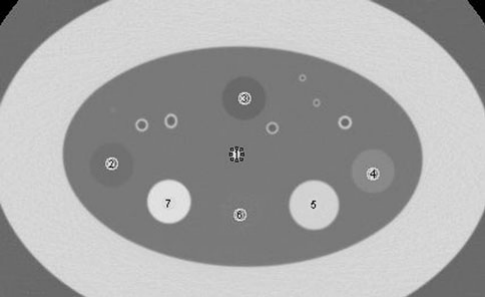
Hounsfield Unit measurements in 7 regions-of-interest (ROI) placed in the phantom. The *white round areas* surrounding the numbers indicate each ROI

The noise simulation software was applied to a COPDGene phantom (CTP699 Lung Phantom: The Phantom Laboratory, Incorporated, Greenwich, NY) scanned with 320 × 0.5 mm detector row CT hardware. Twenty volumetric scans (120 kV, 0.5 s rotation, and 80 × 0.5 mm detector configuration) were acquired, one set with 300 mA and the other using 150 mA. To test the simulation of the 50 % reduced mAs acquisitions, CT noise was added to the sinogram data from the 300 mA acquisition. The sinogram data for the actual 150 mA acquisition and the simulated 150 mA acquisition were identically reconstructed at 0.5 mm slices with a clinical soft tissue kernel (FC13 kernel). The means and SDs of the CT numbers were measured in a 20 mm × 20 mm circular ROI taken in water, air, foam, acrylic and 3 NIST inserts in the phantom (Fig. 1) for both the true and simulated noise reduced data. The COPDGene phantom is surrounded with a uniformity ring (Catphan Uniformity Material Ring) that simulates tissue attenuation.

### Statistical analysis

Data are presented as mean ± SD for the parametric values and median plus interquartile range for the non-parametric values. Regarding the subjective image quality scores and clinical interpretations from two readers before the consensus reading, interobserver agreement was evaluated with Cohen’s kappa test with the following scale: less than 0.20, poor; 0.21–0.40, fair; 0.41–0.60, moderate; 0.61–0.80, good; and 0.81–1.00, excellent agreement. After the consensus reading for the cases with discrepancy scores, the adjudicated scores were used for the Wilcoxon signed-rank test comparing the mean image quality scores between FBP versus FBP50 %, and FBP versus AIDR50 %. The Student’s paired *t* test compared the continuous variables of mean attenuation, and mean SD of the CT number, SNR, and CNR for FBP versus FBP50 % and FBP versus AIDR50 % for both clinical and phantom data.

## Results

### Quantitative image quality of clinical data

There was no significant difference in the CT number among the 5 tissue ROIs for both FBP50 % and AIDR50 %, compared to FBP (Table 2). The image noise of FBP50 % was significantly (*p* < 0.0001) higher than FBP in all tissue areas (Table 3). Image noise of AIDR50 % was lower than the FBP group within the aorta (31.2 ± 8.0 vs. 36.8 ± 12.7, *p* < 0.0001), fat (20.1 ± 5.7 vs. 21.3 ± 7.4, *p* = 0.01), muscle (20.8 ± 5.6 vs. 22.2 ± 7.7, *p* = 0.004) and liver (27.9 ± 6.3 vs. 34.3 ± 10.4, *p* < 0.0001).

**Table 2:** Mean CT number (HU) for the five regions of interest in clinical CTAs

ROI	FBP	FBP50 %	AIDR50 %	FBP versus FBP50 % *p* value	FBP versus AIDR50 % *p* value
Airway	−942.1 ± 38.8	−941.0 ± 39.1	−937.6 ± 38.9	0.85	0.43
Aorta	663.6 ± 206.3	665.0 ± 208.5	661.5 ± 207.7	0.96	0.94
Fat	−102.5 ± 20.2	−103.4 ± 21.0	−102.6 ± 20.5	0.77	0.99
Muscle	61.8 ± 15.1	61.5 ± 15.4	61.2 ± 15.8	0.90	0.79
Liver	71.3 ± 18.4	72.1 ± 19.2	70.5 ± 18.4	0.78	0.77

**Table 3:** Image pixel noise for the five regions of interest in clinical CTAs

ROI	FBP	FBP50 %	AIDR50 %	FBP versus FBP50 % *p* value	FBP versus AIDR50 % *p* value
Airway	28.2 ± 11.4	36.5 ± 14.4	27.7 ± 11.2	<0.0001	0.32
Aorta	36.8 ± 12.7	52.1 ± 19.3	31.2 ± 8.0	<0.0001	<0.0001
Fat	21.3 ± 7.4	29.3 ± 11.4	20.1 ± 5.7	<0.0001	0.01
Muscle	22.2 ± 7.7	31.3 ± 12.7	20.8 ± 5.6	<0.0001	0.004
Liver	34.3 ± 10.4	50.4 ± 17.6	27.9 ± 6.3	<0.0001	<0.0001

The SNR for all tissues within the FBP50 % subjects was significantly lower (*p* < 0.0001) than the FBP group, while the SNR of AIDR50 % was significantly higher than FBP for the aorta (22.6 ± 9.4 vs. 20.2 ± 9.7, *p* < 0.0001) and liver (2.7 ± 1.0 vs. 2.3 ± 1.1, *p* < 0.0001) with no statistical difference for the airway, fat, or muscle (Table 4). The CNR for FBP50 % was also significantly lower (*p* < 0.0001) than FBP (Table 5). However, CNR of aorta to muscle from AIDR50 % was higher than FBP (20.5 ± 9.0 vs. 18.3 ± 9.2, *p* < 0.0001). The CNR of muscle to fat and muscle to airway was not significantly different between the AIDR50 % and FBP subjects (*p* = 0.27 and 0.24, respectively).

**Table 4:** Signal to noise ratio for the five regions of interest in clinical CTAs

ROI	FBP	FBP50 %	AIDR50 %	FBP versus FBP50 % *p* value	FBP versus AIDR50 % *p* value
Airway	38.5 ± 14.5	29.8 ± 11.5	38.3 ± 12.7	<0.0001	0.81
Aorta	20.2 ± 9.7	14.6 ± 7.4	22.6 ± 9.4	<0.0001	<0.0001
Fat	5.4 ± 2.0	4.0 ± 1.6	5.5 ± 1.9	<0.0001	0.20
Muscle	3.1 ± 1.3	2.3 ± 1.0	3.2 ± 1.2	<0.0001	0.38
Liver	2.3 ± 1.1	1.6 ± 0.8	2.7 ± 1.0	<0.0001	<0.0001

**Table 5:** Contrast to noise ratio for the five regions of interest in clinical CTAs

	FBP	FBP50 %	AIDR50 %	FBP versus FBP50 % *p* value	FBP versus AIDR50 % *p* value
Aorta versus Muscle	18.3 ± 9.2	13.2 ± 7.0	20.5 ± 9.0	<0.0001	<0.0001
Muscle versus Fat	8.2 ± 2.9	6.0 ± 2.3	8.4 ± 2.4	<0.0001	0.27
Muscle versus Airway	50.4 ± 17.0	36.9 ± 13.7	51.4 ± 13.7	<0.0001	0.24

### Qualitative image quality of clinical data

The interobserver agreement between the 2 readers was excellent (*κ* = 0.82). For over 88 % (82/93) of subjects, the image quality score among the two readers was identical, and all discrepancies were by 1 point.

**Fig. 2:**
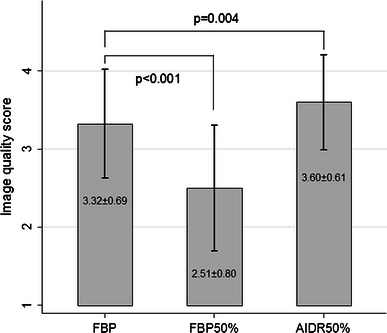
Qualitative image quality score from the filtered back projection (FBP) group, filtered back projection with 50 % dose reduction (FBP50 %) and adaptive iterative dose reduction with 50 % dose reduction (AIDR50 %). -Error bars represent the standard deviations. *p* values from Wilcoxon signed-rank test

For 76 % (71/93) of subjects, the image quality after FBP50 % reconstruction was inferior to the FBP reconstruction group (mean image quality 2.51 and 3.32, respectively; *p* < 0.0001). The image scores were significantly superior (*p* = 0.004) among AIDR50 % reconstructions (mean image quality score 3.60) when compared with FBP (mean image quality score 3.32) (Fig. 2). Of the AIDR50 % reconstructions, 28 % (26/93) had subjectively better image quality than the corresponding FBP reconstruction; for the remaining 67 subjects, the image quality was considered similar.

### Clinical interpretation of data

The overall prevalence of obstructive coronary artery disease was 19 % (18/107). The overall interobserver agreement between the 2 readers was excellent (*κ* = 0.93) with identical reads in 98 % (105/107) for all three reconstruction methods.

**Fig. 3:**
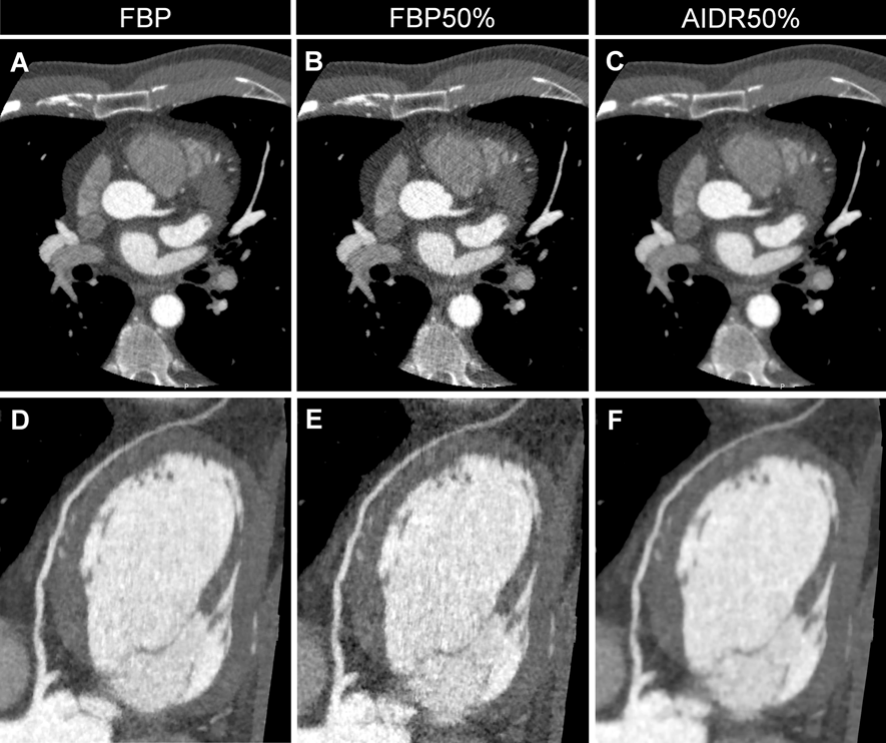
Representative axial (**a**–**c**) and corresponding curved multiplanar reformatted images (**d**–**f**) of the left anterior descending artery from three reconstruction methods performed on one subject (window width/level = 1,200/200). **a** and **d** were reconstructed using conventional filtered back projection (FBP); image noise increases in **b** and **e** that were reconstructed with FBP after a simulated 50 % dose reduction (FBP50 %). **c** and **f** were reconstructed with AIDR3D applied to the raw data after the application of the 50 % simulated noise reduction (AIDR50 %)

**Fig. 4:**
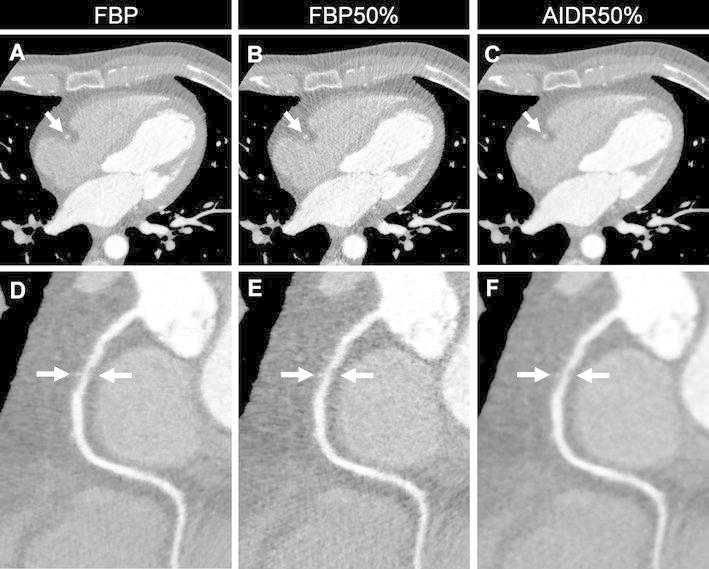
Representative axial (**a**–**c**) and corresponding curved multiplanar reformatted images (**d**–**f**) of the right coronary artery with coronary artery disease (*arrow*) from three reconstruction methods performed on one subject (window width/level = 1,200/200). **a** and **d** were reconstructed using conventional filtered back projection (FBP); image noise increases in **b** and **e** that were reconstructed with FBP after a simulated 50 % dose reduction (FBP50 %). **c** and **f** were reconstructed with AIDR3D applied to the raw data after the application of the 50 % simulated noise reduction (AIDR50 %)

Figure 3 and 4 illustrate representative images for FBP, FBP50 %, and AIDR50 % reconstructions from the same subject. Image noise increased when reconstructing with FBP after a simulated 50 % dose reduction. The AIDR3D reconstruction 50 % dose reduction achieved smoothness of the structural border.

### Phantom study

The mean difference of the absolute CT number between the actual 150 mA acquisition and the simulated 150 mA acquisition was less than 1.6 HU (Table 6). For all ROIs except for ROI5, the difference in mean SD between the two sets of reconstructed images was less than or equal to 0.5 HU and not statistically significant. While very small, the maximum difference of 1.0 HU for ROI5 reached statistical significance (*p* < 0.05).

**Table 6:** Phantom study results

ROI	Mean CT number (HU)			Mean SD		
	Actual 50 mAs	Simulated 50 mAs	Absolute difference	Actual 50 mAs	Simulated 50 mAs	Absolute difference
1	−885.4 ± 0.9	−883.8 ± 0.7	1.6	13.8 ± 0.7	13.5 ± 0.8	0.3
2	−955.7 ± 0.6	−955.1 ± 0.7	0.6	15.0 ± 0.9	14.5 ± 0.7	0.5
3	−1,030.0 ± 0.7	−1,028.5 ± 0.8	1.5	12.6 ± 0.5	12.3 ± 0.5	0.3
4	−704.9 ± 0.6	−704.7 ± 0.6	0.2	16.1 ± 0.8	15.6 ± 0.9	0.5
5	14.7 ± 0.9	15.8 ± 0.8	1.1	17.3 ± 0.7	16.3 ± 0.8	1.0
6	−855.7 ± 0.8	−854.3 ± 0.6	1.3	13.7 ± 0.8	13.3 ± 0.6	0.4
7	138.3 ± 0.9	139.0 ± 0.7	0.7	17.1 ± 0.9	16.7 ± 1.0	0.4

## Discussion

This study is the first to apply a simulated reduced tube current to evaluate wide area detector coronary CT image quality and potential radiation dose reduction in a clinical cohort. These data support the reduction of radiation exposure when AIDR3D is applied to coronary CTA acquisitions, and based on this work, AIDR3D is now in clinical use at both participating institutions.

Radiation dose optimization requires attention to the tradeoff with diagnostic image quality. Idealized studies of these tradeoffs would include multiple acquisitions on the same patient with different exposures [25]. However, individual subject radiation and intravenous contrast loads would be unacceptable. The mathematical addition of CT noise provides the opportunity to directly compare quality among images that depict the same anatomy through simulation of a lower tube current [26]. Noise addition tools have been effectively used to evaluate the effects of dose reduction, primarily outside the heart [27–29]. Both the phantom and the clinical data demonstrate the expected changes in noise based on photon statistics when a 50 % dose reduction is simulated with the noise addition tool (calculated as the product of the noise from FBP and√ 2). The maximum difference of 1.0 HU of noise magnitude in the phantom study, while significant for a single ROI, supports the validity of the noise addition tool. The most likely explanation for the greater variability in clinical data compared to phantom data is the heterogeneity of patient shape, size, and density.

Dose reduction in cardiac CT [21, 22] using iterative reconstruction methods have been extensively investigated [9, 11–13, 17, 18, 30]. One group of studies compares metrics of image quality (e.g. noise levels), without changes in radiation dose, in subjects reconstructed with iterative approaches versus different subjects with images reconstructed using standard FBP. These studies strongly support the high image quality achieved with iterative reconstruction methods.

To evaluate radiation dose optimization, clinical studies again typically use two different patient cohorts: reduced radiation exposure for those subjects reconstructed with iterative methods versus “standard” exposure and estimated radiation dose for those subjects reconstructed with FBP [19, 31, 32]. All based on individual CT platforms, these studies collectively support a 40–50 % dose reduction after the implementation of iterative reconstruction with preservation of image signal, noise, and overall interpretability. Although data from this 320 × 0.5 mm detector row CT study was simulated, the findings suggest that AIDR3D is at first approximation comparable to iterative methods for cardiac CT using other hardware platforms. To our knowledge, only one peer-review publication to date is similar in methodology to our method of simulated dose reduction on the same patient; that study used one of the two x-ray tubes in a dual source system [10].

This study also introduces and tests CT noise addition software for 320 × 0.5 mm detector row CT technology. For cardiac imaging, this scanner enables single heart beat acquisition with temporal uniformity [20, 33] that eliminates cardiac banding artifacts and discontinuities [20] and reduces the patient iodinated contrast burden [34]. To date, there are no prospective wide area detector CT studies that implement AIDR3D with a clinical reference standard. For cardiac imaging, the reference standard could be catheter based angiography [10], clinical outcomes, or both. The current data from simulated images will guide prospective 320 × 0.5 mm CT studies with a 50 % dose reduction. In addition, future work can implement AIDR3D applied to simulated noisy images with clinical interpretation using multi-center data [35, 36] with reference standard imaging and clinical outcomes.

One of the potential drawbacks of iterative reconstructions would be a loss of resolution which is inextricably linked to the reduction of noise. As shown in Fig. 3, the image reconstructed with AIDR3D has “waxy” texture. However, we intend to test our hypothesis that this does not introduce a problem in clinical interpretation. This hypothesis is supported by the superior subjective overall image quality score and by the identical clinical interpretation for obstructive coronary artery disease between AIDR50 % and FBP100 %.

The first main limitation of this study is that the potential dose reduction is simulated and thus, as noted above, prospective studies with reduced exposure will be needed to confirm the clinical benefit of AIDR3D. Second, the subject cohort, although from 2 institutions, is relatively small, and there are no large subject cohorts [37] to compare potential dose reduction between wide area detector CT scanners and earlier CT technology. Third, this study uses noise, SNR, and CNR to characterize and compare the image quality of the dose reduction images compared to the originals. Future work will incorporate the assessment of low contrast detectability with respect to clinical interpretation. In order to quantify the detection of low contrast structure objectively, we are investigating the use of the non-prewhitening matched filter model observer with an addition eye filter to automatically characterize low contrast resolution in phantoms [38]. Finally, regarding the noise addition tool, we recognize some inconsistency in the simulated noise values in air. While these do not impact the current results or conclusion regarding cardiac imaging, further enhancements of the noise software for future applications such as the lung are warranted.

## Conclusion

Using the mathematical addition of CT noise to clinical coronary CT angiograms, the imaging properties of adaptive iterative dose reduction in three-dimensions suggests that the overall image quality can be maintained after a 50 % reduction in radiation dose. Future studies with reduced dose are needed to confirm these findings.
